# Is Adherence to Follow-Up After Bariatric Surgery Necessary? A Systematic Review and Meta-Analysis

**DOI:** 10.1007/s11695-021-05857-1

**Published:** 2022-01-12

**Authors:** Beata M. M. Reiber, Rosalie Barendregt, Ralph de Vries, Sjoerd C. Bruin, Donald L. van der Peet

**Affiliations:** 1 Department of Gastro-Intestinal Surgery, Amsterdam UMC, Location VUmc, de Boelelaan 1118, 1081 HV Amsterdam, the Netherlands; 2 Medical Library, Vrije Universiteit, de Boelelaan 1118, 1081 HV Amsterdam, the Netherlands; 3 Department of Bariatric Surgery, Spaarne Gasthuis, Spaarnepoort 1, 2134 TM Hoofddorp, the Netherlands

**Keywords:** Gastric bypass, Gastric Sleeve, Adherence to follow-up, Short term follow-up, Long term follow-up, Weight loss

## Abstract

The association of adherence to follow-up (FU) after laparoscopic gastric bypass — and gastric sleeve with weight loss (WL) is unclear. The aim of this study was to evaluate this association. Fourteen full text articles were included in the systematic review. Eight studies were included in the meta-analysis concerning FU up to 3 years postoperatively and 3 for the FU between 3 and 10 years postoperatively. Results showed a significant association between adherence to FU 0.5 to 3 years postoperatively and percentage excess WL (%EWL) but did not demonstrate a significant association between FU > 3 years postoperatively and total WL (%TWL). In conclusion, adherence to FU may not be associated with WL and therefore stringent lifelong FU in its current form should be evaluated.

**Supplementary Information:**

The online version contains supplementary material available at 10.1007/s11695-021-05857-1.

## Introduction

Severe obesity has increased rapidly in the Netherlands with a current estimate of more than 10% of adults with a BMI > 40 kg/m2 (CBS 2011). This is known to be associated with increased mortality [1]. Bariatric surgery has become an important treatment option for severe obesity and its long lasting effects on both weight loss (WL) and comorbidities have been proven [2–8]. Current guidelines emphasize the importance of strict follow-up (FU) postoperatively for several reasons, in order to timely recognize and treat postoperative adverse effects that can occur long after surgery and to monitor WL or weight regain as loss to FU has been associated with insufficient WL [6–10]. Adherence to this strict FU however remains remarkably challenging and consequently multiple studies have pointed out disappointing adherence to FU (40–62% after 2 years) with varying patient related risk factors for this attrition [11, 12]. This multifactorial challenge in addition to a significant influx of patients due to a rise in the burden of disease results in logistical challenges within hospitals and again raises the question if postoperative follow-up is really necessary. This systematic review and meta-analysis therefore aimed to evaluate the influence of adherence to follow up on postoperative WL after laparoscopic Roux-en-Y gastric bypass (LRYGB) and laparoscopic sleeve gastrectomy (LSG).

## Methods

### Search Strategy

A literature search was performed based on the preferred reporting items for systematic reviews and meta-analyses (PRISMA) statement (www.prisma-statement.org).

To identify all relevant publications, systematic searches were conducted in the bibliographic databases PubMed, Embase.com, and Web of Science from inception up to July 12, 2020, in collaboration with a medical librarian. The following terms were used (including synonyms and closely related words) as index terms or free-text words: “Gastric Bypass,” “Post-bariatric Surgery,” “Weight Loss Surgery,” “Lost to Follow-Up,” “No Show,” “Drop Out.” The references of the identified articles were searched for relevant publications. Duplicate articles were excluded. All languages were accepted in the initial process. The full search strategies for all databases can be found in Appendix A/Supplementary material.

### Selection Process

Two reviewers (BR and RB) independently screened all potentially relevant titles and abstracts for eligibility. If necessary, the full text article was checked for the eligibility criteria. Differences in judgement were resolved through a consensus procedure. Studies were included if they met the following criteria: (i) clinical study, (ii) describing LRYGB or LSG, (iii) used standardized methods of measuring postoperative weight loss, (iv) compared postoperative weight loss between adherent and non-adherent groups, and (v) was written in English. Studies were excluded if they (i) were not written in English (ii), only described surgical procedures other than LRYGB or LSG or if the results of all procedures were only pooled into one group, (iii) were not conducted in adults, and (iv) belong to certain publication types: editorials, letters, legal cases, interviews, etc.

### Data Assessment

Two reviewers (BR and RB) independently evaluated the methodological quality of the included full text papers using the Newcastle–Ottawa quality assessment scale (NOS) for cohort studies and case–control studies [13] and the Jadad score for randomized controlled trials.

### Data Extraction

Baseline characteristics were extracted for the adherent and non-adherent groups of each study separately. If only data concerning the total group were presented, those were extracted. The following characteristics were extracted from each study: number of patients, percentage of females, mean age and standard deviation, mean BMI prior to surgery and standard deviation, the follow-up duration at which the latest weight loss results were obtained, and the operation type. From each study, definitions of adherence and/or non-adherence were collected. Postoperative weight loss in either percentage excess weight loss (%EWL), percentage total weight loss (%TWL), or both at the longest FU duration was collected where available. Articles were included in the meta-analysis when both mean and standard deviation values were published or could be calculated from median and interquartile ranges. Unpublished data were sought by contacting original authors, extracted from studies in which the same database was used, or computed based on previously published formulas [14]. Meta-analysis was performed in two groups: short term (0–3 years postoperatively) and long term (> 3 years postoperatively). Review manager (version 5.4.1, the Cochrane Collaboration, 2020) was used, performing a random effect model with *I*^2^ expressing heterogeneity. Subgroup analysis was performed to explore heterogeneity where necessary.

## Results

### Systematic Review

**Fig. 1:**
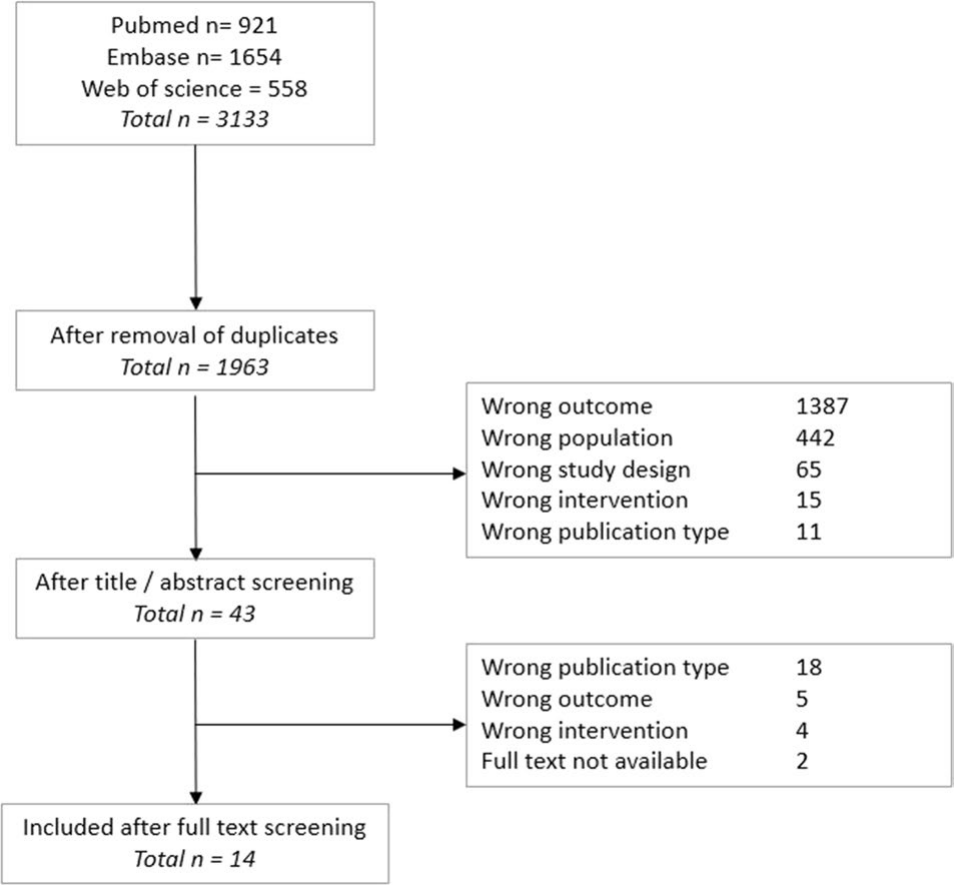
Flowchart depicting the selection process of articles for review

**Fig. 2:**
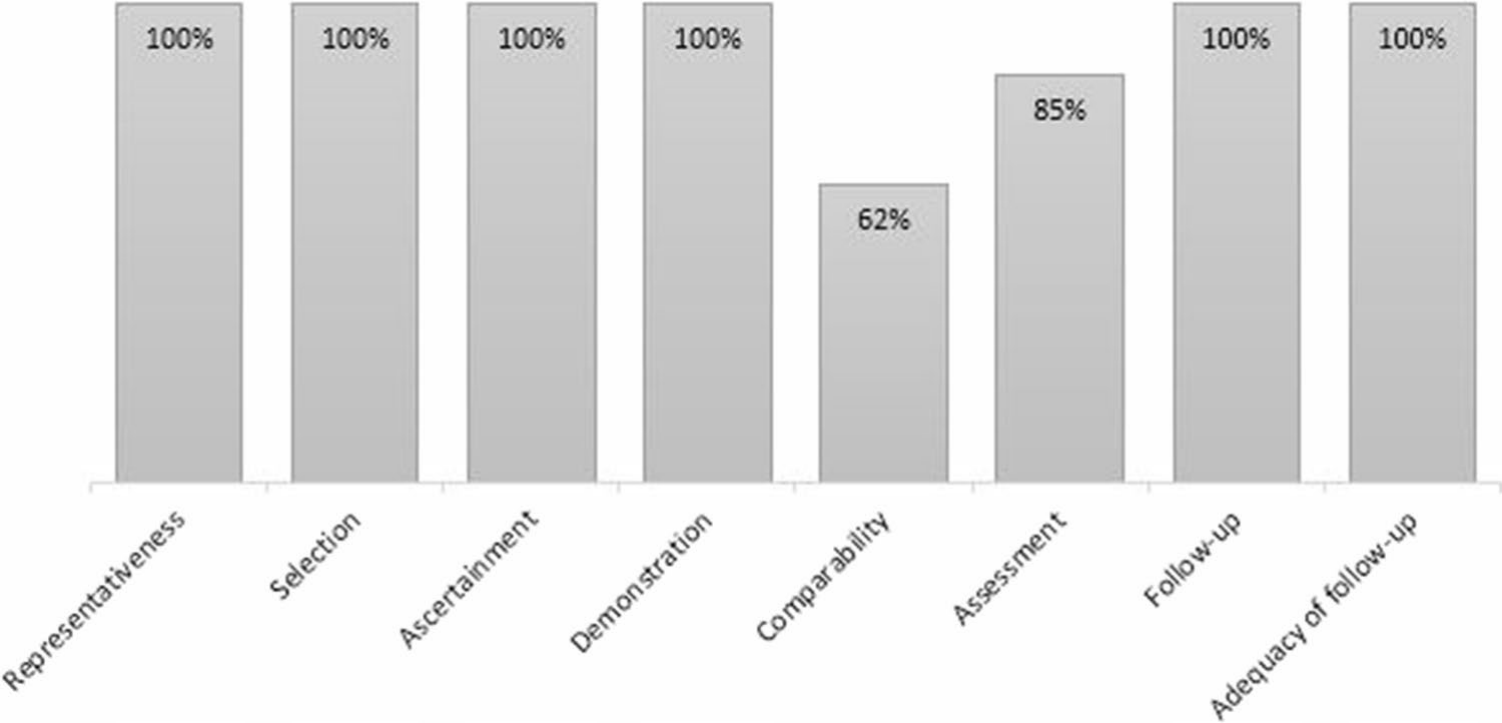
Quality assessment of cohort studies using the Newcastle–Ottawa Scale (NOS) assessment, depicting the percentage of studies that complied with the separate items of the NOS

The literature search generated a total of 3133 references. After the screening of title and abstracts and consequently the remaining full text articles, 14 full text articles were included. The flow chart of the search and selection process is presented in Fig. 1. As all included studies were either prospective- (*n* = 5) and retrospective (*n* = 9) cohort studies, quality assessment of all included articles was performed according to the NOS checklist. The mean number of point scores per item is depicted in Fig. 2. All available baseline characteristics, duration of FU and type of operation (LRYGB or LSG), are depicted in Table 1 per group (i.e., adherent and non-adherent) per study. Table 2 demonstrates how adherence and non-adherence were defined in each study separately. Table 3 shows the type and amount of WL being reported by the authors and whether the adherence to FU was positively associated with a higher WL including *p*-value if reported. Out of 10 studies describing 0–3 year FU, 6 studies found a positive association of adherence to FU with postoperative WL [10, 15–24]. Out of 4 studies describing 3–10 year FU, 2 studies found this positive association [25–28].

**Table 1:** Baseline characteristics of the adherent – and non-adherent groups per study

	Adherent patients				Non-adherent patients					
	Number of patients	% female	Mean age (SD) or range	Mean start BMI (SD) or range	Number of patients	% female	Mean age (SD) or range	Mean start BMI (SD) or range	FU duration in months	Operation type
Compher et al., 2012^15^	32	78%	46.9 (10.1)	54.5 (10.2)	28	64%	46.7 (10.9)	49.2 (9.4)	24	LRYGB
Goldenshluger et al., 2018^25^	83	68%*	39.9 (11.2)*	42.9 (4.5)*	95	*	*	*	48	LSG
Gould et al., 2007^16^	34	91%	47 (10)	51 (7)	51	78%	42 (10)	50 (7)	36	LRYGB
Harper et al., 2007^17^	57	88%	38 (9)	48 (6)	42	79%	40 (10)	49 (6)	12	LRYGB
Jennings et al., 2013^18^	180	59%	47.3 (NS)	52.1 (NS)	59	62%	46.0	52.3	24	LRYGB
Keren et al., 2011^19^	83	65%	40.4 (9.7)	44.5 (6.9)	36	58%	39.3 (11.3)	44.8 (5.5)	30	LSG
Lujan et al., 2020^26^	101	64.6%	51.4 (10.6)	40.1 (8.7)	193	59.9%	45.8 (10.9)	44.9 (6.8)	60	LRYGB
Mehaffey et al., 2016^27^	151	NS	41.4 (NS)	53.1 (NS)	500	NS	40.6 (-)	53.5	120	LRYGB**
McVay et al., 2013^20^	405	77.5%	45.8 (11.5)	48.4 (7.9)	133	72.9%	43.4 (10.7)	48.6 (8.4)	6	LRYGB
Shen et al., 2004^21^	53	75%	40.8 (10.8)	47.0 (7.8)	62	84%	41.9 (8.4)	48.7 (5.2)	12	LRYGB
Shilton et al., 2019^22^	139	72.7%	46.7 (NS)	47.6 (7.4)	45	75.6%	44.0 (NS)	46.6 (6.8)	24	LRYGB + LSG
Spaniolas et al., 2016^23^	38,613	78.5%	48 (37–55)	46.1 (41.8–52.3)	12,468	79.6%	48 (39–56)	46.1 (41.8–52)	12	LRYGB + LSG
Vidal et al., 2013^28^	216	81%*	45.4 (9.0)	44.8 (5.9)	46	*	41.7 (8.6)	44.2 (4.6)	72	LRYGB + LSG
Welch et al., 2011^24^	75*	85.3%*	43.8 (10.9)*	49.8 (6.9)*	*	*	*	*	30	LRYGB

^*^These characteristics were only mentioned for the entire population and not per subgroup. **51.5% of the population underwent open Roux-en-Y gastric bypass. *FU*, follow-up*; LRYGB*, laparoscopic Roux-en-Y gastric bypass; *LSG*, laparoscopic gastric sleeve; *NS*, not stated; *SD*, standard deviation

**Table 2:** Definition of adherence and non-adherence per study

	**Adherence**	**Non-adherence**
Compher et al., 2012^15^	Patients attended the 1 year FU appointment	Patients missed the 1 year FU appointment
Goldenshluger et al.,2018^25^	Patients attended ≥ 6 meetings	Patients attended ≤ 5 meetings
Gould et al., 2007^16^	Patients attended every FU 3–4 years postoperatively	Patients did not attend FU either after 1 or 2 years postoperatively
Harper et al., 2007^17^	Patients attended the 1 year FU appointment	Patients did not attend the 1 year FU appointment
Jennings et al., 2013^18^	Patients attended all FU appointments	Patients missed either 1 (single default) or > 1 (poor attendees) FU appointments
Keren et al., 2011^19^	Patients attended all FU appointments	Patients missed the FU appointments
Lujan et al., 2020^26^	Patients attended all FU appointment	Patients were lost to FU before 1 year postoperatively
Mehaffey et al., 2016^27^	Patients attended all yearly FU appointments	Patients missed the FU appointments after 2 years postoperatively
McVay et al., 2013^20^	Patients attended 3 or 4 FU appointments	Patients attended ≤ 2 appointments postoperatively
Shen et al., 2004^21^	Patients attended ≥ 3 out of 5 FU appointments (LRYGB group)	Patients attended ≤ 3 FU appointments (LRYGB group)
Shilton et al., 2019^22^	Patients missed < 4 FU appointments	Patients missed ≥ 4 FU appointments
Spaniolas et al., 2016^23^	Patients completed follow-up	Patients missed the 3- and/or 6-month visit
Vidal et al., 2013^28^	Patients attended FU appointments ≥ 6 months	Patients missed any FU appointment ≥ 6 months postoperatively
Welch et al., 2011^24^	Not clearly stated	Not clearly stated

*FU*, follow-up; *LRYGB*, laparoscopic Roux-en-Y gastric bypass

**Table 3:** Weight loss measures and association of adherence with WL

	FU duration in months	Type of WL measured	Adherent group %EWL / %TWL / other	Non-adherent group %EWL / %TWL / other	Significant association adherence with WL / *p*-value	Study included in meta-analysis
Compher et al., 2012^15^	24	EWL / TWL	70.7%/35.7% / -	53.4%/18.7% / -	Yes / -	Yes
Goldenshluger et al.,2018^25^	48	TWL	- / 29.18% / -	- / 31.28% / -	No / 0.160	Yes
Gould et al., 2007^16^	36	EWL	74% /—/ -	60% /—/ -	Yes / < 0.05	Yes
Harper et al., 2007^17^	12	EBWL	- /—/ 76%*	- /—/ 65%*	Yes / < 0.003	Yes
Jennings et al., 2013^18^	24	EWL	66.9% /—/ -	59.5% /—/ -	No / 0.06	No
Keren et al., 2011^19^	30	EWL / EBMIL	80.01%/—/ 82.08%**	72.53%/—/ 74.88%**	Yes / < 0.001	Yes
Lujan et al., 2020^26^- LRYGB	60	EWL / TWL	72.21% / 26.85% / -	62.72%/ 27.63% / -	Yes / < 0.05 EWL / NS TWL	Yes
Lujan et al., 2020^26^- LSG	60	EWL / TWL	77.48% / 25.42% / -	38.87% / 17.22% / -	Yes / < 0.001 EWL / < 0.01 TWL	Yes
Mehaffey et al., 2016^27^	120	TWL / EBMIL	- / 26.3% / 52.2%**	- / 28.3% / 52.8%**	No / 0.73 TWL / 0.36 EBMIL	Yes
McVay et al., 2013^20^	6	EWL / EBMIL	48.4% /—/ -	43.9% /—/ -	Yes / < 0.05	Yes
Shen et al., 2004^21^	12	EWL	67.6% /—/ -	66.1% /—/ -	No / -	Yes
Shilton et al., 2019^22^	24	EWL / TWL	70.9% / 32.4% / -	60.7% / 26.0% / -	No / 0.12 EWL / 0.029 TWL	Yes
Spaniolas et al., 2016^23^	12	TWL / EBWL	- / 34% / 65% *	- / 33% / 63% *	Yes / < 0.001 TWL / < 0.001 EBWL	Yes
Vidal et al., 2013^28^	72	EWL	-	-	Yes	No
Welch et al., 2011^24^	30	EWL / BMIL	-	-	No	No

*BMI*, body mass index; *BMIL*, percentage BMI Loss; *EBMIL*, excess BMI loss; *EBWL*: excess body weight loss; *EWL*: excess weight loss; *FU*: follow-up; *NS*: not significant; *TWL*: total weight loss; *WL*: weight loss; *-*, not stated. *EBWL was reported. **EBMIL was reported

### Studies Excluded for Meta-Analysis

Jennings et al. [18] described a cohort of 227 patients (180 adherent and 47 non-adherent patients). At 1 year FU, there was a significant difference in percentage excess weight loss (%EWL) in favor of adherent patients (65.5% versus 59.5%, *p* 0.01). The statistical significance was however lost at 2-year FU (*p* 0.06). Unfortunately, the study could not be included into the meta-analysis as appropriate variance values were missing. For similar reasons, the study of Welch et al. was excluded from the meta-analysis. Welch et al. [24] performed a broader assessment of factors influencing %EWL 2–3 years after surgery. After performing backward regression analysis however adherence to and the frequency of FU were no significant predictors of %EWL. Due to the dichotomous manner of reporting outcomes (i.e., sufficient EWL versus insufficient EWL), the study of Vidal et al. [28] was excluded from this meta-analysis as well. The study included 217 adherent and 46 non-adherent patients with a maximum FU period of 8 years. Non-adherence was significantly higher in the group demonstrating insufficient weight loss (< 50% EWL).

### Meta-Analysis

#### Short-Term FU

**Fig. 3:**
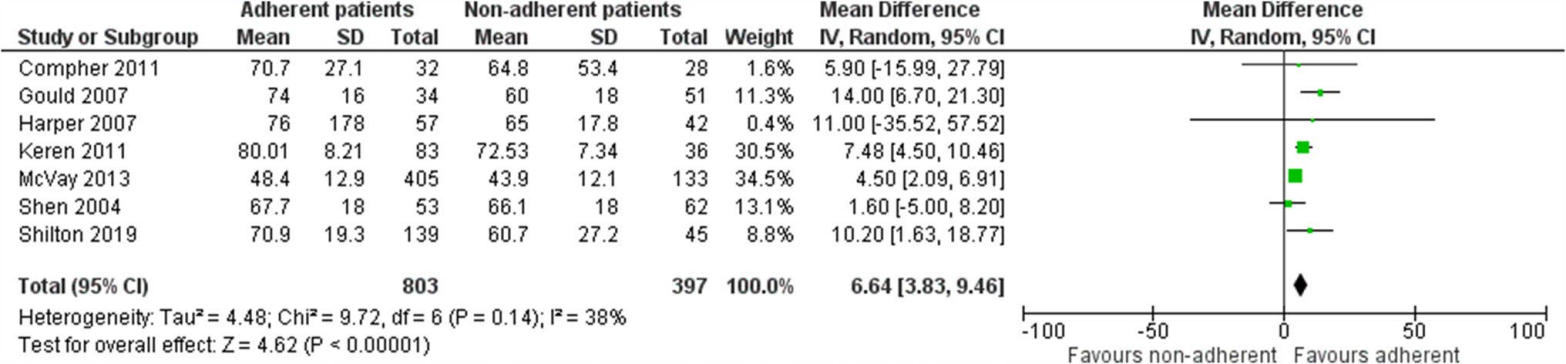
Meta-analysis of excess weight loss between adherent and non-adherent groups 0.5 to 3 years postoperatively

Eight of the eleven studies describing short-term FU were included. The forest plot is shown in Fig. 3. A random effect model was used and the outcome was calculated into standardized mean difference. The latter is due to the fact that 2 studies used the percentage excess body weight loss (%EBWL = (initial BMI − BMI at FU) / (initial BMI: 25 kg/m^2^) × 100%), whereas all others used the percentage excess weight loss (%EWL = (initial weight, weight at FU) / (initial weight, ideal weight based on BMI 25 kg/m^2^) × 100%). The pooled effect size of adherence to short-term FU was 6.23%. The initial *I*^2^ was 82%. However, when the most obvious outliers were removed for the subgroup analysis, *I*^2^ became 0% and the pooled effect size was 8.83%. The outliers were identified as follows: McVay et al. due to its very short-term FU (6 months), Shen et al. due to a looser definition of adherence to FU (more than 3 appointments), and Spaniolas et al. due to its remarkably large sample size (51,081 patients).

#### Long-Term FU

**Fig. 4:**
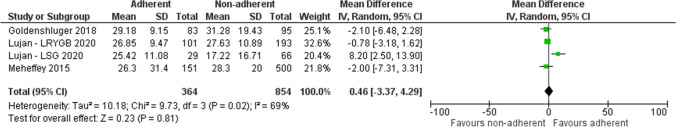
Meta-analysis of total weight loss between adherent and non-adherent groups more than 3 years postoperatively

Three of the four studies describing long-term FU were included. The forest plot is shown in Fig. 4. All outcomes were reported in %TWL; hence, a random effect model with mean difference as an outcome was calculated. As Lujan et al. reported the results for LSG and LRYGB separately, data were entered accordingly. The pooled effect size of adherence to long-term FU was 0.46%. The initial *I*^2^ was 69%. However, when the most obvious outlier was removed for the subgroup analysis, *I*^2^ became 0% and the pooled effect size − 1.21%. In this analysis, the outlier was the LSG subgroup of Lujan et al. with an evidently much lower %TWL in the non-adherent group; 17.22 (SD 16.71) whereas all other studies described a %TWL over 25.

## Discussion

As this study reported short- and long-term FU results separately and in different WL outcomes (i.e., excess weight loss versus total weight loss), they should be interpreted and discussed separately.

### Short-Term FU Results

The current study concerning FU up to 3 years postoperatively shows a significant association between adherence to FU and postoperative excess weight loss. This study therefore updates and confirms the meta-analysis of Kim et al. that only explored the association of adherence to short-term FU 1 year after LRYGB with postoperative excess weight loss [29]. Several factors of concern possibly leading to bias in this part of the meta-analysis remain. The most important challenge was the fact that nearly all studies used the percentage excess weight loss as the sole outcome measure and consequently no total weight loss values were available. It has become increasingly well known that %EWL is too heavily influenced by the initial BMI which may well have biased this part of the analysis [30, 31]. Aside from its role in %EWL, initial BMI and initial age separately are well-known independent factors influencing postoperative WL results. However, these factors were both generally similar for adherent and non-adherent patients in all included studies [32–34]. Four of the included short-term studies in this meta-analysis demonstrated 7–13% more female patients in the adherent groups. Previous studies have shown that male patients tend to show more weight regain or less weight loss [32]. This may have influenced the results leading to a stronger association between adherence to FU and postoperative WL. Taking the above into careful consideration, the results concerning short-term FU should be interpreted in combination with previous studies that showed that early detection of nutrient deficiencies and late (surgical) complications is desirable in order to minimize long-term negative effects. Furthermore, remission of comorbidities has been shown to be associated with %EWL [6–8, 35, 36]. It therefore seems safe to assume that the first 3 years after bariatric surgery; adherence to FU could be advised but may well be debated.

### Long-Term FU results

The long-term FU (more than 3 years postoperatively) results of the present study show no association between adherence to FU and total weight loss. These results extend the previous meta-anaysis of Kim et al. and faced a few different challenges than the short-term FU part of the study. The 4 cohorts studied by Lujan et al. (adherent versus non-adherent for LRYGB and LSG separately) demonstrated important and statistically significant differences between the groups; non-adherent patients after LSG had an initial BMI of 49.18 whereas adherent LSG patients had an initial BMI of 40.24 (*p* < 0.001). This may well explain the differences in %TWL outcome after 5 years as a higher initial BMI is a well-known risk factor for inadequate weight loss [33, 34, 37]. Similarly, non-adherent LRYGB patients had a significantly higher BMI and were significantly younger than adherent LRYGB patients. This may have led to an underestimation of the %TWL of the non-adherent group (%TWL 26.85% for adherent patients and 27.63% for non-adherent patients 5 years after surgery). However, after removing this study from the subgroup analysis, the pooled effect size remained similar.

From this study, it can therefore be concluded that long-term FU might not be necessary to maintain weight loss after LRYGB and LSG but more studies are needed in order to draw a safe evidence-based conclusion. Consequently, the remaining argument substantiating the indication of lifelong FU after bariatric surgery may be that of nutrient deficiencies. Previous studies have shown that these deficiencies may reveal despite adherence to daily multivitamins on the long term and may inflict long-term harm in patients [38, 39]. However, Higa et al. did show that nutrient deficiencies were as common in patients coming into the office for their FU appointment as in patients who were reporting by phone. This could suggest that lifelong physical FU may not be necessary in order to timely detect nutrient deficiencies [38]. Aside from nutrient deficiencies, gastroesophageal reflux disease (GERD) has been suggested as an indication for lifelong FU after LSG. A recent study proposed standardized endoscopic surveillance after LSG in order to timely detect GERD sequelae, especially as these were also detected in patients without any symptoms. In the future, this may be an argument to continue follow up in LSG patients [40].

The authors of this study however suggest that long-term FU in a bariatric center of excellence is not necessary in terms of weight loss and emphasize that stringent lifelong follow-up in its current form should be evaluated.

This study should be interpreted in the light of its limitations. The most important limitation is the heterogeneity in the definition of (non) adherence between studies. Two studies had high expectations of their patients already defining them as non-adherent after missing one appointment; however, most studies had looser requirements. Having missed only one appointment may have led to an underestimation of the effect of non-adherence on weight loss. The authors therefore suggest a uniform definition of adherence and non-adherence to be used in future research, due to the wide variety of frequency and form of FU; the authors suggest non-adherence should be defined as not attending one single appointment a year and consequently not having undergone any weight- and nutrient deficiency control. Additionally, the importance of reporting weight loss in a unified matter should be stressed as various studies were excluded from the meta-analysis as they could not be pooled. Lastly, the presence of comorbidities was not described in all included studies, which may have influenced the results as for example the presence of diabetes mellitus is associated with decreased postoperative WL [37].

## Conclusion

Based on this systematic review and meta-analysis, it can be concluded that more than 3 years postoperatively, there may not be an influence of adherence to FU on WL after LRYGB and LSG. Up to 3 years postoperatively, this influence remains debatable as well. Consequently, despite extensive protocols concerning stringent lifelong FU, the authors of this study suggest that (long-term) follow-up in a bariatric center of excellence may not be necessary in terms of weight loss and emphasize that the follow-up in its current form should be evaluated.

## Supplementary Information

Below is the link to the electronic supplementary material.

Supplementary file1 (DOCX 16 KB)
