# Supplementary material for: Is Adherence to Follow-Up After Bariatric Surgery Necessary? A Systematic Review and Meta-Analysis
Source: Obes Surg. 2022 Jan 12;32(3):904–11. doi: 10.1007/s11695-021-05857-1 (PMC8866276; doi:10.1007/s11695-021-05857-1)
Supplement: Supplementary file 1 — Supplementary file1 (DOCX 16 KB) [file 11695_2021_5857_MOESM1_ESM.docx]

**Appendix A / Supplementary material**

**PubMed Session Results (12 July 2020)**

| Search | Query | Items found |
| --- | --- | --- |
| #3 | **#1 AND #2** | 921 |
| #2 | **"Patient Acceptance of Health Care"[Mesh] OR "Lost to Follow-Up"[Mesh] OR Lost to follow-up*[tiab] OR Loss to follow-up*[tiab] OR attrition*[tiab] OR adherence to follow up[tiab] OR no show*[tiab] OR noshow*[tiab] OR dropout*[tiab] OR drop-out*[tiab] OR nonadheren*[tiab] OR noncomplian*[tiab] OR non-adheren*[tiab] OR non-complian*[tiab] OR postoperative follow-up*[tiab] OR post-operative follow-up*[tiab] OR non-attend*[tiab] OR nonattend*[tiab] OR patient absen*[tiab] OR non-appear*[tiab] OR nonappear*[tiab] OR follow-up complian*[tiab] OR follow-up adheren*[tiab] OR follow-up attend*[tiab] OR follow-up appear*[tiab] OR follow-up loss*[tiab]** | 227,593 |
| #1 | **"Bariatric Surgery"[Mesh:noexp] OR "Gastric Bypass"[Mesh] OR "Anastomosis, Roux-en-Y"[Mesh] OR gastric bypass*[tiab] OR Roux-en-Y[tiab] OR sleeve*[tiab] OR postbariatric surg*[tiab] OR post-bariatric surg*[tiab] OR weight loss surg*[tiab] OR weight reduction surg*[tiab]** | 34,576 |

**Embase.com Session Results (12 July 2020)**

| Search | Query | Items found |
| --- | --- | --- |
| #3 | **#1 AND #2** | 1,654 |
| #2 | **'patient attitude'/de OR 'patient attendance'/exp OR 'patient dropout'/exp OR 'refusal to participate'/exp OR 'treatment interruption'/exp OR 'loss to follow up'/exp OR 'Lost to follow-up*':ab,ti,kw OR 'Loss to follow-up*':ab,ti,kw OR attrition*:ab,ti,kw OR 'adherence to follow up':ab,ti,kw OR 'no show*':ab,ti,kw OR noshow*:ab,ti,kw OR dropout*:ab,ti,kw OR 'drop-out*':ab,ti,kw OR nonadheren*:ab,ti,kw OR noncomplian*:ab,ti,kw OR 'non-adheren*':ab,ti,kw OR 'non-complian*':ab,ti,kw OR 'postoperative follow-up*':ab,ti,kw OR 'post-operative follow-up*':ab,ti,kw OR 'non-attend*':ab,ti,kw OR nonattend*:ab,ti,kw OR 'patient absen*':ab,ti,kw OR 'non-appear*':ab,ti,kw OR nonappear*:ab,ti,kw OR 'follow-up complian*':ab,ti,kw OR 'follow-up adheren*':ab,ti,kw OR 'follow-up attend*':ab,ti,kw OR 'follow-up appear*':ab,ti,kw OR 'follow-up loss*':ab,ti,kw** | 215,352 |
| #1 | **'bariatric surgery'/de OR 'gastric bypass surgery'/exp OR 'gastric banding'/exp OR 'sleeve gastrectomy'/exp OR 'Roux Y anastomosis'/exp OR 'gastric bypass*':ab,ti,kw OR 'Roux-en-Y':ab,ti,kw OR sleeve*:ab,ti,kw OR 'postbariatric surg*':ab,ti,kw OR 'post-bariatric surg*':ab,ti,kw OR 'weight loss surg*':ab,ti,kw OR 'weight reduction surg*':ab,ti,kw** | 70,430 |

**Web of Science (Core Collection) Session Results (12 July 2020)**

| Search | Query | Items found |
| --- | --- | --- |
| #3 | **#1 AND #2** | 558 |
| #2 | **TS=("Lost to follow-up*" OR "Loss to follow-up*" OR attrition* OR "adherence to follow up" OR "no show*" OR noshow* OR dropout* OR "drop-out*" OR nonadheren* OR noncomplian* OR "non-adheren*" OR "non-complian*" OR "postoperative follow-up*" OR "post-operative follow-up*" OR "non-attend*" OR nonattend* OR "patient absen*" OR "non-appear*" OR nonappear* OR "follow-up complian*" OR "follow-up adheren*" OR "follow-up attend*" OR "follow-up appear*" OR "follow-up loss*")** | 108,210 |
| #1 | **TS=("gastric bypass*" OR "Roux-en-Y" OR sleeve* OR "postbariatric surg*" OR "post-bariatric surg*" OR "weight loss surg*" OR "weight reduction surg*")** | 39,802 |
